# DNA and the origins of life in micaceous clay

**DOI:** 10.1016/j.bpj.2022.08.032

**Published:** 2022-09-20

**Authors:** Helen Greenwood Hansma

**Affiliations:** 1 Department of Physics, University of California, Santa Barbara, Santa Barbara, California

## Abstract

Reproducible imaging of DNA by atomic force microscopy was a useful predecessor to Ned Seeman’s DNA nanotechnology. Many of the products of DNA nanotechnology were imaged in the atomic force microscope. The mica substrate used in this atomic force microscopy research formed the inspiration for the hypothesis that micaceous clay was a likely habitat for the origins of life. Montmorillonite clay has been a successful substrate for the polymerization of amino acids and nucleotides into peptides and DNA oligomers in research on life’s origins. Mica and montmorillonite have the same anionic lattice, with a hexagonal spacing of 0.5 nm. Micas are nonswelling clays, with potassium ions (K^+^) holding the crystal sheets together, providing a stable environment for the processes and molecular complexes needed for the emergence of living cells. Montmorillonite crystal sheets are held together by smaller sodium ions (Na^+^), which results in swelling and shrinking during wet-dry cycles, providing a less stable environment. Also, the cells in all types of living systems have high intracellular K^+^ concentrations, which makes mica a more likely habitat for the origins of life than montmorillonite. Finally, moving mica sheets provides mechanical energy at the split edges of the sheets in mica “books.” This mechanical energy of mica sheets, moving open and shut, in response to fluid flow, may have preceded chemical energy at life’s origins, powering early prebiotic processes, such as the formation of covalent bonds, the interactions of molecular complexes, and the budding off of protocells before the molecular mechanism of cell division had developed.

## Significance

Atomic force microscopy is a valuable technique for imaging DNA and other biological molecules by raster scanning a tip back and forth across a sample to generate a three-dimensional “map” of the surface and the molecules on it. Atomic force microscopy has been useful for imaging novel DNA and RNA structures on the surface of mica, which is atomically flat. Mica is also a possible place where the origins of life might have taken place. One of the advantages of mica is that its mineral sheets are held together by potassium ions, and potassium ions are also present in large amounts in the cells of living organisms of all types. The origin of life is a major scientific puzzle.

## Introduction

Ned Seeman was too young to die (or, Nadrian Seeman, as he was named in ∼99% of his publications in Google Scholar)! He and I were born within days of each other. I remember the excitement in the lab when we heard about his 1991 *Nature* paper on the synthesis of a DNA cube (1), which started the field of DNA nanotechnology (2).

I first met Ned Seeman at SUNY Albany at Ramaswamy Sarma’s 21^st^ Conversation in June 1999. Jeff Gelles and I gave talks at the session on “Imaging at the molecular level,” Gelles on “Mechanisms of Transcription and Transcription Regulation in Single RNA Polymerase Molecules,” and I on “New Insights from Atomic Force Microscopy of DNA” (3,4). Later that afternoon, Laura Landweber, Erik Winfree, and Ned Seeman gave talks at the session on “DNA and RNA computing,” Landweber on “Computing with RNA,” Winfree on “Algorithmic Self-Assembly of DNA,” and Seeman on “Two Dimensions and Two States in DNA Nanotechnology” (3,5,6). Seeman’s publication about this talk begins with a quote from an English poet: “… a flock of crazy prophets, that by staring at a crystal can fill it with more fancies than there are herrings in the sea. … Alfred Noyes” (6).

**Figure 1:**
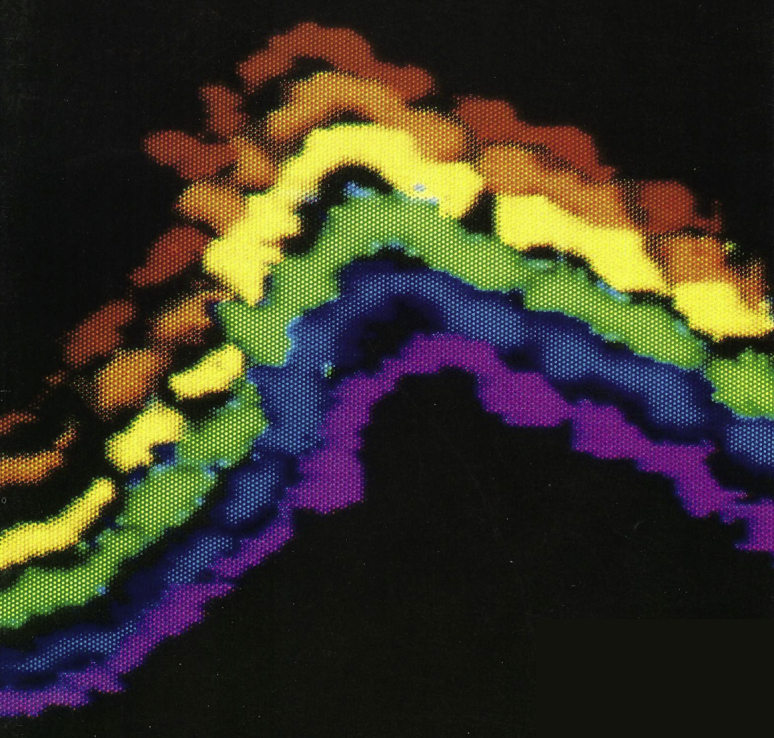
Reproducible imaging of plasmid DNA under liquid with the atomic force microscope (7). Nearly identical images were recorded during 15 min of continuous scanning. Image width is ∼100 nm. This was a useful development for future AFM imaging of structures made by DNA nanotechnology. Computer art by S.D. Hansma. Rainbow DNA image is from the *Science* magazine cover of May 22, 1992.

**Figure 2:**
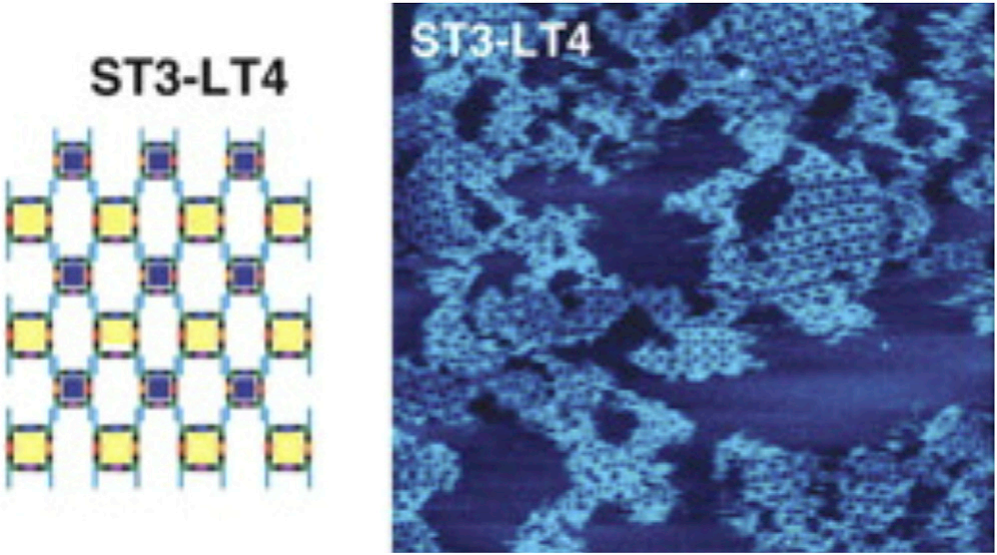
Basket-weave pattern of RNA constructs. Design (*left*) and 1 *μ*m AFM image (*right*). ST3-LT4, small and large tectosquares. Tectosquares are each made from 2 RNA hairpins that interact via kissing loop interactions (9). To see this figure in color, go online.

Much work on DNA and RNA nanotechnology used atomic force microscopy (AFM) imaging, which benefitted from “Reproducible Imaging and Dissection of Plasmid DNA under Liquid with the Atomic Force Microscope” (Fig. 1) (7). Two projects using AFM imaging were “Design and self-assembly of two-dimensional DNA crystals” in 1998 (8) and “Building Programmable Jigsaw Puzzles with RNA” in 2004 (9) (Fig. 2).

The most memorable paper, for me, was Rothemund’s “Folding DNA to create nanoscale shapes and patterns.” (10) This paper was published during another epoch of my life, bringing to mind where I was when I first saw his DNA smiley face on the cover of *Nature*. “More than just a smiley face,” reported Bethany Halford in 2012. “Researchers struggle to find practical applications for nanoscale patterning technique” (11).

## DNA nanotechnology, molecular machines, and the origins of life

DNA nanotechnology and research on the origins of life both seek biomimetic systems. Research on the origins of life requires one to imagine different ways of accomplishing the things that are done by living organisms today. The “RNA World” is one outcome of this research, in which ribozymes carry out enzymatic reactions that are carried out by proteins in living systems.

**Figure 3:**
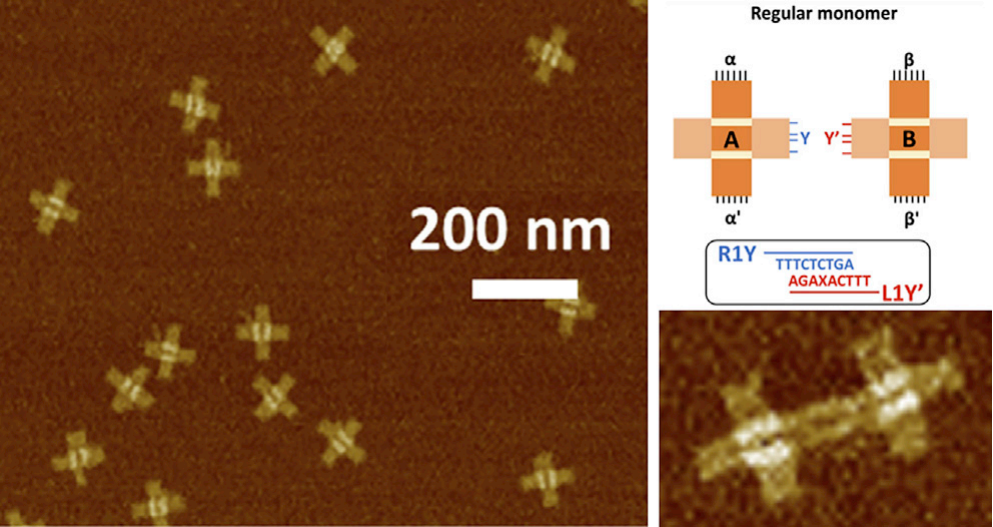
Monomers and dimers, AFM images, and diagram from “Mutations in artificial self-replicating tiles: A step toward Darwinian evolution” (14). To see this figure in color, go online.

Research in DNA nanotechnology has produced aspects of a “DNA World” in which molecular machines are made from DNA instead of proteins. Ned Seeman and his colleagues pioneered this research. As a first development in this direction, DNA tile motifs were developed that could recognize and bind complementary tiles in a preprogrammed fashion (12). A few years later, self-replication and evolution were developed in DNA origami rafts (13). Near the end of his life, Seeman and collaborators introduced mutations into self-replicating DNA origami to better understand molecular evolution and to evolve new materials and devices with useful properties (14) (Fig. 3).

In other DNA nanotechnology research, the hydrodynamic forces in bursting bubbles were measured via the fragmentation of DNA nanotubes as the bubbles burst (15). Self-replicating nonbiological materials are the goal of research on colloidal particles with sticky ends of single-stranded DNA (16). Nonenzymatic replication is a goal of research in which DNA-containing crystals are mechanically fragmented (17). In another type of DNA nanotechnology, silver nanoclusters of reproducible sizes—and many colors—form through interactions with DNA molecules of different sequences (18).

### DNA and the origin of life in clay

Life’s origin in clay has a long history, extending back to creation stories. The RNA World is considerably newer (19). One question about the origins of life is the following: “Why do the nucleotides in life’s RNA and DNA form 3′-5′ linkages between phosphate and sugar residues?” Alternate linkages are 2′-5′ and pyrophosphate linkages, both of which form more rapidly in solution than the biological 3′-5′ linkages (20). Early work by Ferris, Ertem, and others showed that the biological 3′-5′ linkages form preferentially on the clay mineral montmorillonite (20,21).

### Hypothesis for the origin of life between mica sheets

**Figure 4:**
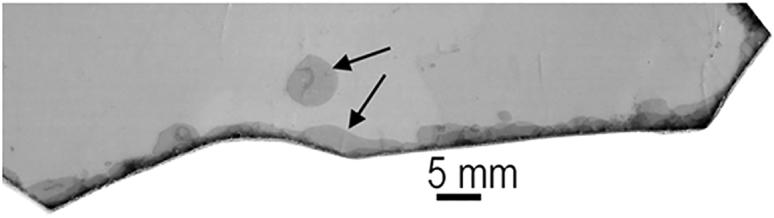
Micas are nonswelling clay minerals that can separate at the edges of their sheets. Photograph of muscovite mica, showing a bubble (*top arrow*) and separation at the edges of the mica (*bottom arrow*). Bubbles are common, even in “high-grade” micas such as this one.

Mica is a clay mineral. Mica is also a substrate for imaging DNA by AFM, and it may have been a substrate for the origins of life. Micas, such as muscovite and biotite, are nonswelling clay minerals whose anionic mineral sheets are held together by potassium ions (K^+^) (22). In contrast, montmorillonite is a swelling clay mineral whose anionic mineral sheets are held together by sodium ions (Na^+^). Swelling clay minerals swell and shrink during wet-dry cycles, causing the clay mineral sheets to move apart and together. Micas do not swell and shrink, but the edges of the sheets in mica “books” can separate and move open and shut in response to wetting and drying (Fig. 4).

What are the advantages of micaceous clay over montmorillonite clay? Micaceous clay has at least two advantages. One advantage is that nonswelling mica provides a more stable environment for the formation of prebiotic molecules and the development of prebiotic processes. Second, in all types of living organisms, the intracellular environment is higher in K^+^ than in Na^+^ (23). Maintaining high intracellular K^+^ requires cells to invest large amounts of energy (24,25). Why would life start to evolve in an environment high in Na^+^ and then “decide” to create an energy-intensive gradient of high intracellular K^+^?

How did the hypothesis arise that life originated between mica sheets? This is described in “Granny says life evolved between the mica sheets” on livescience.com (26). The original inspiration came when I had not a scientific thought in my head (though a math colleague at the National Science Foundation thought one could not be looking through a dissecting microscope, even in one’s home, without a scientific thought in their head). The National Science Foundation later changed the title of the article, on their website, to “Researcher says life evolved between the mica sheets,” thus removing two categories of diversity: “old” and “woman” (27).

**Figure 5:**
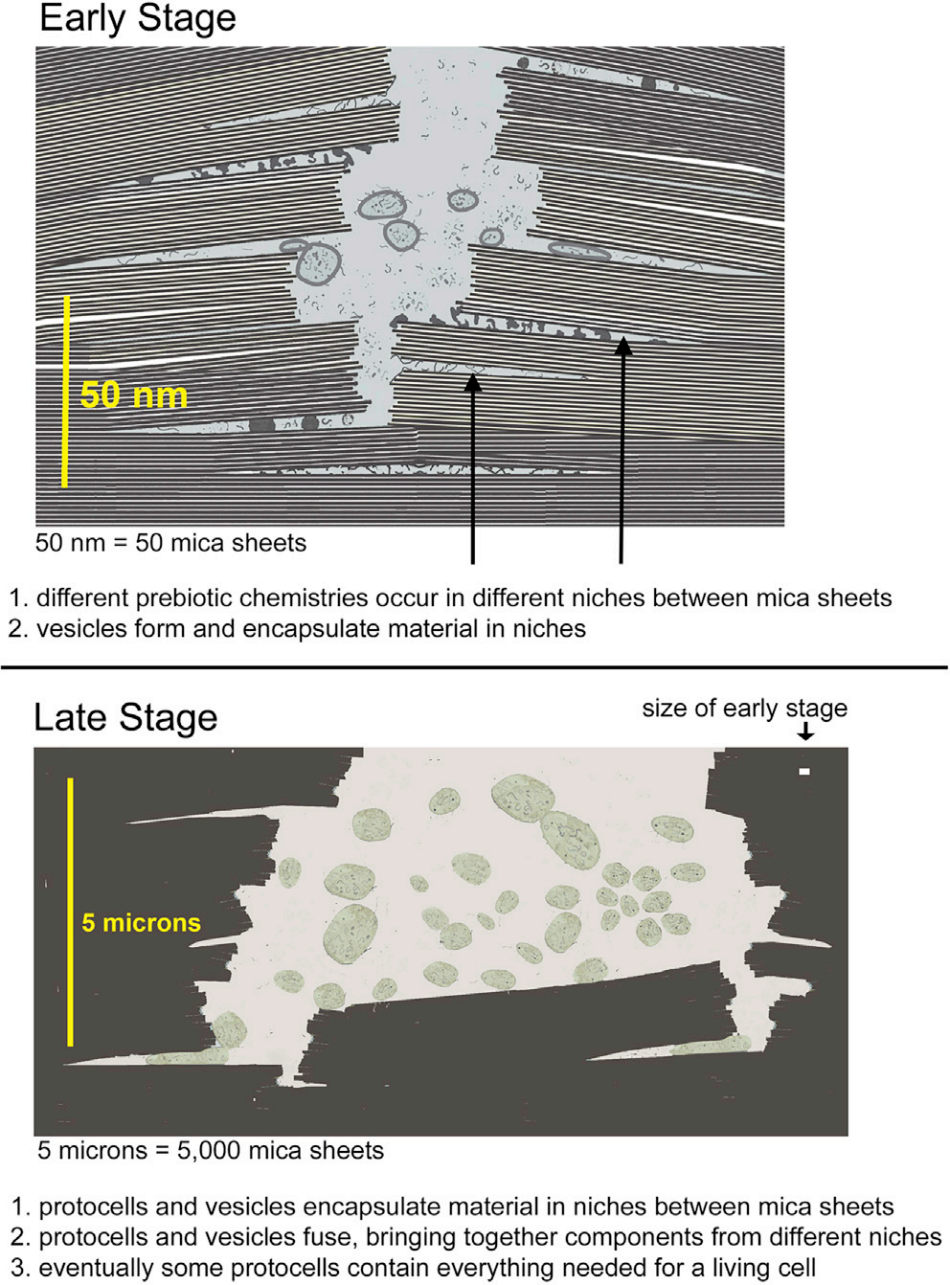
Origin of life between mica sheets. Early stage: nanometer-scale diagram of how the early stages of life might have originated between biotite mica sheets. Niches within the biotite sheets provide partially enclosed spaces for the molecular evolution of the different processes that are essential for life. Vesicles form, encapsulating molecules and molecular complexes in the niches. Late stage: micron-scale diagram of how living cells might have developed within biotite mica. Protocells in the aqueous environment encapsulate prebiotic molecular aggregates in the niches between mica sheets. Mechanical energy from moving mica sheets can bleb off protocells, as seen in the lower left corner of the figure. Eventually, a living cell capable of self-reproduction could be produced. To see this figure in color, go online.

How might life have originated between mica sheets? (Fig. 5) Life must have originated in a place hospitable for all the components of living systems. This hospitable place is hypothesized to be within mica (28,29,30,31,32). Membranes were not necessary for the early stages of protolife. Instead, membraneless organelles, or coacervates, formed through liquid liquid phase separation (33). Ribosomes, for example, are similar to tiny membraneless organelles (32).

Eventually, life emerged as cells within membranes. The transition from mica to membranes is straightforward because vesicles and membranes were ubiquitous on the early Earth, as described in “The Lipid World” (34) and many other publications such as these (35,36,37). These lipids would have wrapped themselves over and around the prebiotic components within mica. The resulting vesicles would have fused, bringing together different components of prebiotic life, producing protocells that continued to fuse—and divide—resulting eventually in protocells containing all the components of living systems. Survivors among these protocells were the earliest living cells, which seeded Earth with life (or Mars, which also has biotite, if life first emerged on Mars (38,39)). These were the progenote (40), or the last universal common ancestor (LUCA), of all life today (41).

As the reviewers noted, scenarios for the origins of life contain improbable, missing, or miraculous steps. These steps are being filled in, bit by bit, by ongoing research. For example, amino acids and nucleic acids have been produced from cyanide, ammonia, and carbon dioxide (42).

Micas are an ideal habitat for life’s origins. Their clay mineral anionic lattice has a spacing of 0.5 nm, equal to the spacing of anionic phosphate groups on extended single-stranded nucleic acids such DNA and RNA. As in living cells, exchangeable inorganic cations bridge the mica sheets and the nucleic acids. The spaces between mica’s mineral sheets would have provided “reaction chambers” where the components necessary for life could evolve—peptides, ribozymes, autocatalytic and metabolic cycles, and, eventually, ribosomes, replication of information, and the other components of the simplest living cells. Vast amounts of time would have passed. Life also imitates mica in many ways (Table 1 in (28)).

The hypothesized early and late stages of life’s origins between mica sheets are diagrammed and described in Fig. 5, at the nanometer scale in the early stage and at the micron scale in the late stage. Open systems like the niches between mica sheets may have been needed early in life’s origins to facilitate free exchanges (43).

Biomolecules of many types bind to mica (44,45). Many biomolecular and enzymatic processes have been observed on mica in the AFM. These include DNA transcription by RNA polymerase (46), DNA degradation by DNase I (47), the assembly of DNA complexes with RNA polymerase (48), and lysozyme activity (49). RNA also polymerizes on mica, nonenzymatically, from nucleotide monophosphates of A, U, G, and C (50,51).

### How much K^+^ is there between mica sheets?

**Figure 6:**
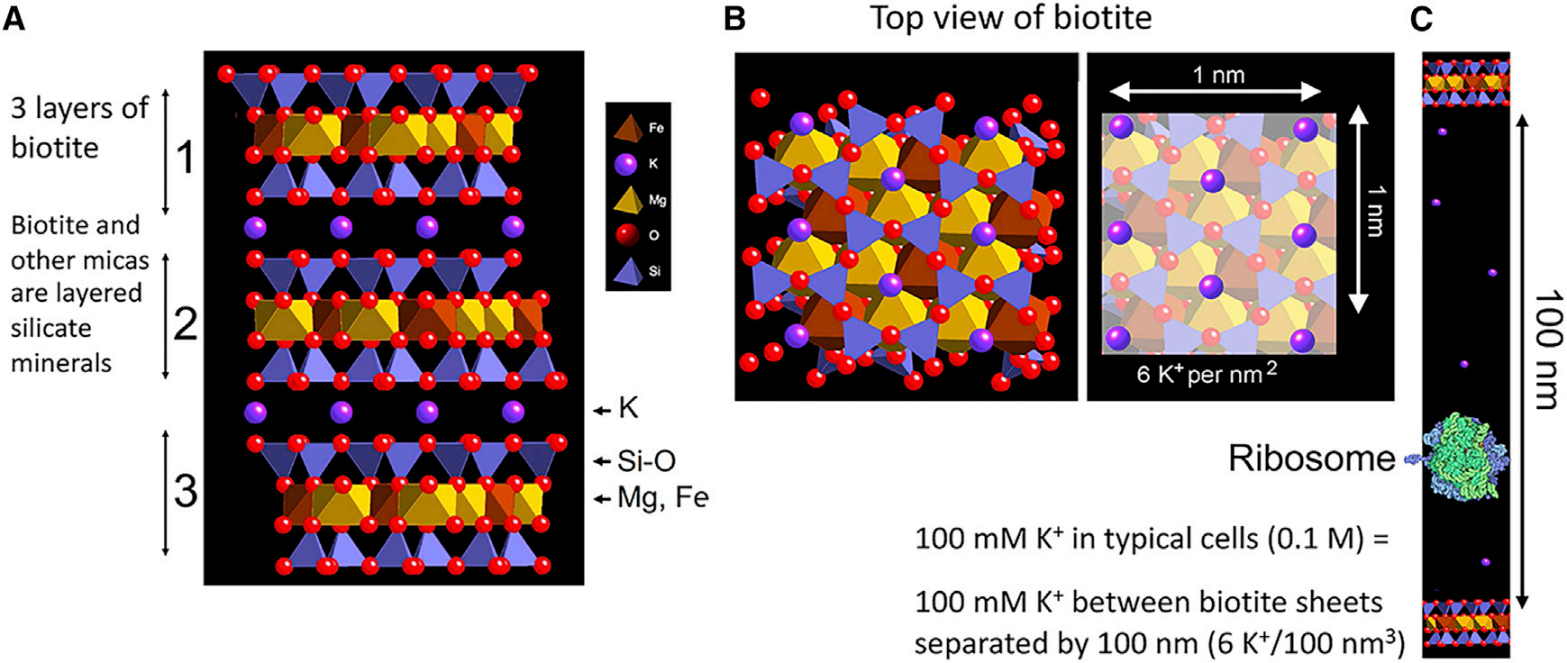
There is a large amount of [K^+^] between mica sheets (28,52). When mica sheets are separated by 100 nm, the [K^+^] is at a typical physiological concentration of ∼100 nm. (*A*) Structure of the black mica, biotite. Side view of three biotite sheets, labeled 1, 2, and 3. (*B*) Top view of 1 nm^2^ biotite, with K^+^ highlighted in the right-hand image, showing that there are six K^+^ per nm^2^ between mica sheets. (*C*) Scale model of biotite sheets at a separation of 100 nm, with bacterial ribosome, showing relative sizes of bacterial ribosome and space between mica sheets where [K^+^] = 100 mM. Biotite mica might be better than muscovite mica for life’s origins, due to the ferrous iron in biotite for redox reactions (28). This figure was prepared with CrystalMaker X software, v.10.6.4, CrystalMaker Software (Oxfordshire, UK). To see this figure in color, go online.

There is so much K^+^ between mica sheets that if the mica sheets are separated to a distance of 100 nm, the K^+^ concentration, [K+], is 100 mM, comparable to the [K+] in living cells (23) (Fig. 6). On the scale of a bacterial ribosome, a separation of 100 nm appears to be large enough for the earliest life to evolve (Fig. 6 *C*). 100 mM is an average of [K+] between mica sheets separated by 100 nm; K^+^ will be entering the space on the inside of the split mica sheets, and K^+^ will be leaving at the outer side of the split mica sheets, giving higher and lower [K+], respectively.

### Energy from mica sheets

**Figure 7:**
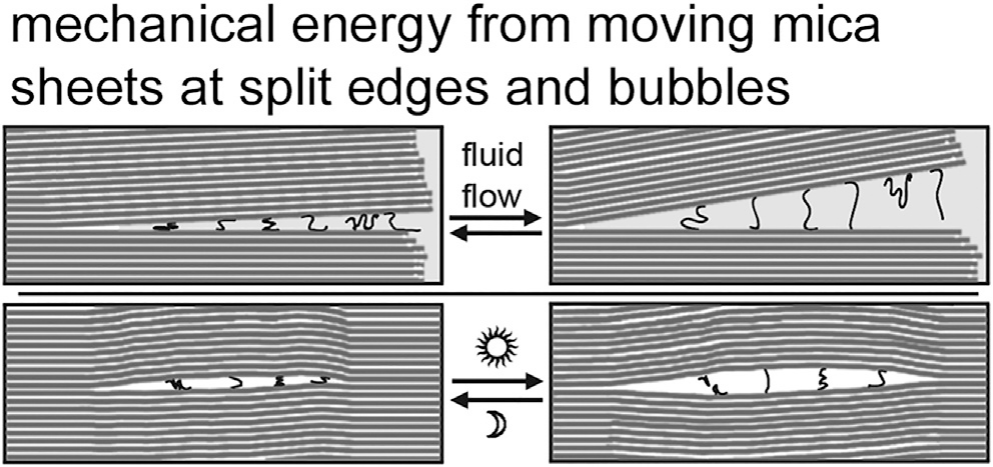
Moving mica sheets generate mechanical energy for mechanochemistry. Diagrams of mechanical forces between mica sheets show stretching and compressing of polymers due to fluid flow at the edges of the mica sheets (*top diagrams*) and forces between mica sheets due to heat pumps in a mica bubble (*bottom*). This mechanical energy can be used to synthesize prebiotic molecules, stretch and compress polymers (as shown in the diagram), or bleb off protocells (31). Seven mica sheets provide enough force to form a covalent bond in air when moved to a distance of 0.1 nm (28,53).

Moving mica sheets can produce endless mechanical energy for the origins of life (Fig. 7) (29). This mechanical energy, or work, can be used for mechanochemistry to make and break chemical bonds, when mica sheets move, open and shut, in air or fluid. This mechanical energy can also be used to induce molecular interactions and to cause the budding off of vesicles and protocells in a primitive form of cell division.

Mechanical energy may have preceded chemical energy at life’s origins (29). In lipids, mechanical energy, and forces, also have an ancient origin. A new paper on forces from lipids shows that “what is true for *E. coli* is true for the elephant.” (54)

Polymers form in clay during wet-dry cycles, as discussed by Ross and Deamer in “Prebiotic Oligomer Assembly: What Was the Energy Source?” (55). Similarly, polymers form during wet-dry cycles on mica sheets, as observed for RNA on mica (50,51).

### How big do the mica sheets need to be?

**Figure 8:**
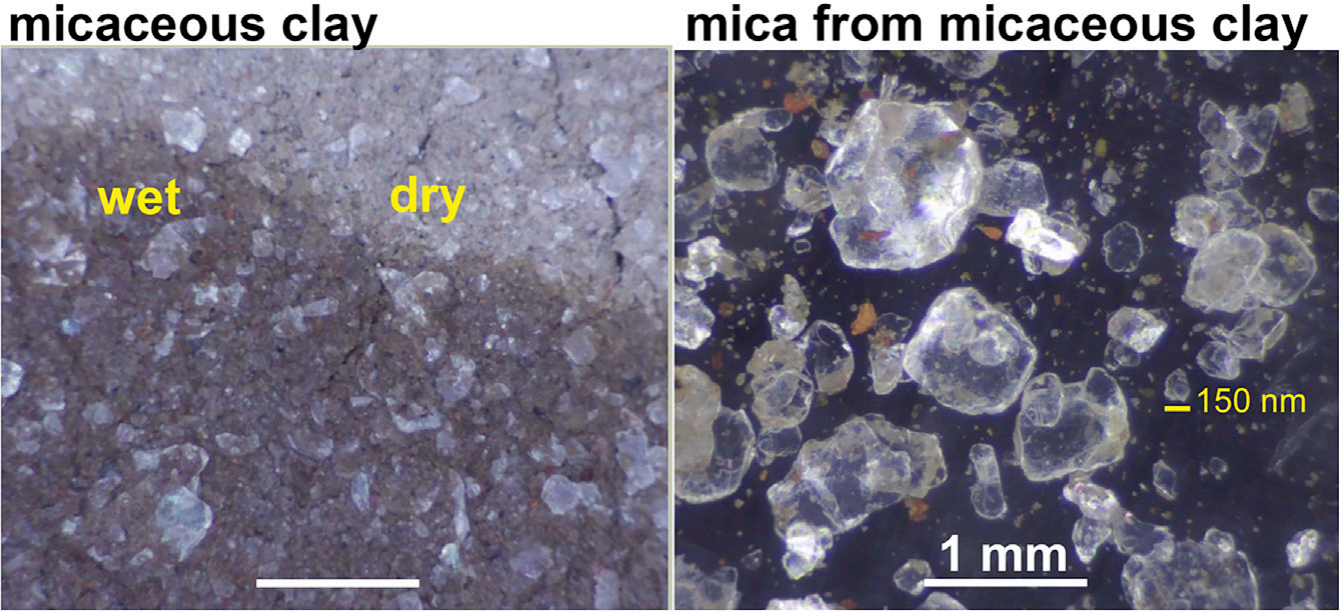
“Mica red” micaceous clay from New Mexico Clay store. Left: clay in a wet-dry cycle with pale reflecting pieces of mica. Right: mica (*translucent pieces*) and a few clay particles washed from the micaceous clay. One small mica piece has a diameter of 150 nm (*yellow scale bar*). White scale bars are both 1 mm. To see this figure in color, go online.

The tiny mica fragments in micaceous clay are large enough for mechanochemistry (Fig. 8), given that nanometer-sized movements are adequate to bring molecules together to form covalent bonds (28). There would not have been a lot of micaceous clay at life’s origins, but mica is old enough to have been a habitat where life originated (56).

The clay environment of micaceous clay would provide many benefits for life’s origins. One such benefit is the free exchanges of molecules and molecular complexes between mica and the larger diverse environment of the surrounding nonmicaceous clay—and the environment beyond the clay. Research on life’s origins in clay is extensive and includes these publications in the last decade (57,58,59,60,61,62).

**Figure 9:**
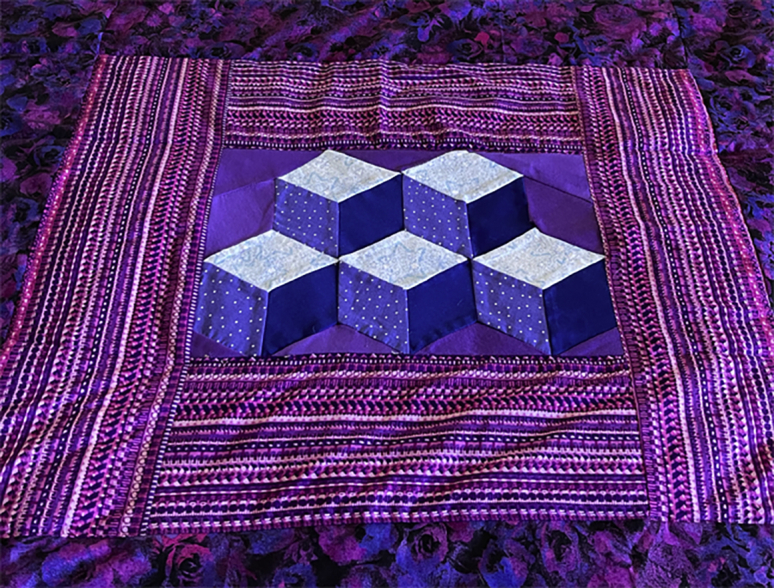
Escher-inspired quilt by H. Hansma. To see this figure in color, go online.

A final tribute to Ned Seeman and his enthusiasm for Escher is the photo of an Escher-inspired quilt in Fig. 9. Seeman wrote about art as a stimulus for structural DNA nanotechnology (63). For example, when thinking about branched DNA with more than just four arms, Seeman remembered the Escher woodcut *Depth* (64).
